# First description of the females of *Qinorapalaqinlingana* Chou & Wang, 1995 (Lepidoptera, Lycaenidae) from Shaanxi and Sichuan Provinces, western China

**DOI:** 10.3897/BDJ.12.e117061

**Published:** 2024-03-15

**Authors:** Sixun Ge, Wen-Hao Sun, Yang Yang, Li-Li Ren, Shao-Ji Hu

**Affiliations:** 1 College of Forestry, Beijing Forestry University, Beijing, China College of Forestry, Beijing Forestry University Beijing China; 2 Water Resources Research Institute of Shandong Province, Jinan, China Water Resources Research Institute of Shandong Province Jinan China; 3 Shandong Province Key Laboratory of Water Resources and Environment, Jinan, China Shandong Province Key Laboratory of Water Resources and Environment Jinan China; 4 Room 515, No.10 Building, Xiaoguanbei Lane, Anwai Street, Chaoyang District, Beijing, China Room 515, No.10 Building, Xiaoguanbei Lane, Anwai Street, Chaoyang District Beijing China; 5 Institute of International Rivers and Eco-security, Yunnan University, Kunming, China Institute of International Rivers and Eco-security, Yunnan University Kunming China

**Keywords:** taxonomy, Sichuan, Oriental Region

## Abstract

**Background:**

The family Lycaenidae is a widely distributed and species-rich group with approximately 5300 described species. The rare genus *Qinorapala* Chou & Wang, with *Q.qinlingana* Chou & Wang as its type species was established as monotypic. In the original description, *Q.qinlingana* was described from a male holotype; the female remained unknown. To date, the genus is only recorded from the Qinling Mountains (Shaanxi and Gansu Provinces). In this study, two female specimens, from Shaanxi Province and western Sichuan Province (bordering Yunnan Province) are described and illustrated for the first time.

**New information:**

Female specimens of *Q.qinlingana* from Shaanxi and Sichuan are described for the first time. The species' distribution is updated and a distribution map is provided.

## Introduction

The family Lycaenidae (common name: gossamer-winged butterflies) is a widely distributed and species-rich group with about 5300 described species (Pierce et al. 2002). As the second largest family of Papilionoidea, this group includes many model animals and also several pests, which jointly play important roles in diverse ecosystems (Sharma and Sharma 2021). Most adults of the group are small and brightly coloured with metallic iridescence on the uppersides (Shou et al. 2006).

The monotypic genus *Qinorapala* Chou & Wang includes only the rare type species *Q.qinlingana* Chou & Wang. In the original description, *Q.qinlingana* was described from a male holotype; females were unknown. To date, the genus is only recorded from the Qinling Mountains (Shaanxi and Gansu Provinces).

In this study, two female specimens from Shaanxi Province and western Sichuan Province (bordering Yunnan Province) are described and illustrated for the first time. Kimura 2-parameter (K2P) distances between mitochondrial COI barcode region sequences were calculated. A distribution map of the species is provided.

## Materials and methods

### Morphological comparison

The specimens from Sichuan Province were photographed using an interchangeable lens digital camera Sony A7M2 digital SLR camera with a Laowa 100 mm macro lens. Final plates were prepared in Adobe Photoshop CC (adobe.com). To study the female genitalia, the abdomens were taken from the specimens and placed into a 1.5 ml microcentrifuge tube, then soaked in 10% potassium hydroxide solution at room temperature for about 24 hours. After dissection, the genitalia were then transferred to 80% glycerol for 12 h to render them transparent.

### Molecular phylogenetic analysis

DNA extraction, PCR amplification and sequencing follows Ge et al. (2023). The forward primer LepF (5'-ATTCAACCAATCATAAAGATATTGG-3') and reverse primer LepR (5'-TAAACTTCTGGATGTCCAAAAAATCA-3') employed in Lukhtanov et al. (2009) were used in this study. The phylogeny was reconstructed using the Neighbour-Joining method by Mega11, with 500 replications of bootstrap to test the phylogeny (Tamura et al. 2021). Four specimens of *Q.qinlingana* were selected as ingroups, with three of them newly sequenced in this study (two males from Shaanxi Province and a female from Sichuan), the remaining one being extracted from Kawahara et al. (2023). Sequences of outgroup taxa were selected amongst the mostly closely-related species with COI data on GenBank according to the genus-level phylogeny of Kawahara et al. (2023). All new sequences were deposited in GenBank (BioProject PRJNA967499: accession numbers OR825799-OR825801).

## Data resources

Three specimens of *Q.qinlingana* employed in phylogenetic analysis were from Si-Xun Ge's collection and Shi-Yu Tong's collection (a male from Shaanxi Province belongs to Shi-Yu Tong's collection, another male from Shaanxi and the female from Sichuan belongs to Si-Xun Ge's collection). New sequences were deposited in GenBank (BioProject PRJNA967499: accession numbers OR825799-OR825801).

## Taxon treatments

### Qinorapala

Chou & Wang, 1995

25EAF320-4F0B-5E29-B2C9-F0A186BBB551

Qinorapala qinlingana Chou & Wang, 1995Chou and Wang 1995

#### Diagnosis

Forewing with veins M_1_ and R_4+5_ stalked, male with a hair tuft on underside along dorsum. Hind-wing with a short tail at vein CuA_2_ and a scent brand in space Sc+R_1_, tornal lobe poorly-developed. Male genitalia with valve not conjoined ventrally.

#### Distribution

West China (Shaanxi, Gansu, Sichuan).

### Qinorapala qinlingana

Chou & Wang, 1995

E76DA4C0-FC6F-5436-8E6B-CF12026BA331

#### Materials

- **Type status:** Other material. **Occurrence:** sex: female; preparations: photograph; occurrenceID: 4047D57B-B010-52AB-811E-00D9FD0C0D0F; **Taxon:** scientificName: *Qinorapalaqinlingana*; order: Lepidoptera; family: Lycaenidae; genus: Qinorapala; taxonRank: species; **Location:** higherGeography: West China; country: China; countryCode: CN; stateProvince: Shaanxi; verbatimLocality: Jialing River Source Scenic Spot; verbatimElevation: 2800 m; **Identification:** identifiedBy: Jian Luo; **Event:** eventTime: 2011; year: 2011
- **Type status:** Other material. **Occurrence:** sex: female; preparations: photograph; DNA extract; associatedSequences: GenBank: OR825799.1; occurrenceID: 9B833B39-427D-598D-9A13-96C22C88272C; **Taxon:** scientificName: *Qinorapalaqinlingana*; order: Lepidoptera; family: Lycaenidae; genus: Qinorapala; specificEpithet: qinlingana; taxonRank: species; **Location:** country: China; countryCode: CN; stateProvince: Sichuan; county: Derong; locality: 8–10 km south of Bendu township (along the National Highway 215); verbatimElevation: 2500-3000 m; **Identification:** identifiedBy: Sixun Ge; **Event:** year: 2023; month: 4
- **Type status:** Other material. **Occurrence:** individualCount: 2; sex: 2 males; lifeStage: adult; preparations: photograph DNA extract; associatedSequences: GenBank: OR825800.1; OR825801.1; occurrenceID: 08E7368E-2E02-5B86-82E8-B3CD7A961634; **Taxon:** scientificName: Qinorapalaqinlingana; family: Lycaenidae; genus: Qinorapala; **Location:** country: China; countryCode: CN; stateProvince: Shaanxi; county: Ningshan; locality: Huangguan Township; verbatimElevation: 1300 m; **Identification:** identifiedBy: Sixun Ge; **Event:** samplingEffort: Sweep net; eventDate: 1 May 2020; year: 2020; month: 5; day: 1; **Record Level:** basisOfRecord: PreservedSpecimen

#### Description

**Figure 1.**
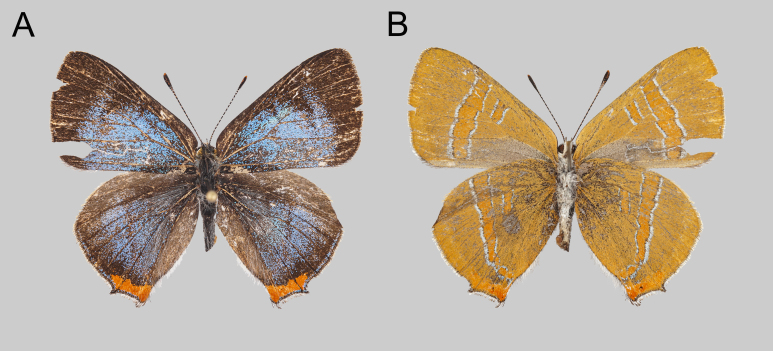
Female of *Qinorapalaqinlingana* Chou & Wang, 1995 collected 8-10 km south of Bendu township (along National Highway 215), Derong County, Sichuan, China: upperside (**A**) and underside (**B**).

**Female from Sichuan Province** (Fig. 1) : Length of forewing 16.5 mm. Upperside (Fig. 1): Fore- and hind-wings with distinct sky-blue iridescence, forewing with veins brownish, partly suffused with blue scales; post-discal band brownish, weakly developed; median band faint and narrow, extended to space Cu_1_. Dark brownish border broadening at apex rather developed, from costal to tornal angle. Hind-wing ground colour as in forewing with costal margin and outer margin brownish; outer margin with distinct sky-blue marginal band. Tails extremely short; orange tornal spot bright-coloured, distinctly developed in spaces Cu_2_, Cu_1_ and M_1_. Underside (Fig. 1): Both wings pale orange coloured, post-discal band and median band pale tangerine with narrower whitish margins. Tornus of hind-wing light orange-coloured, similar to upperside with a tiny black spot in space 2.

**Figure 2.**
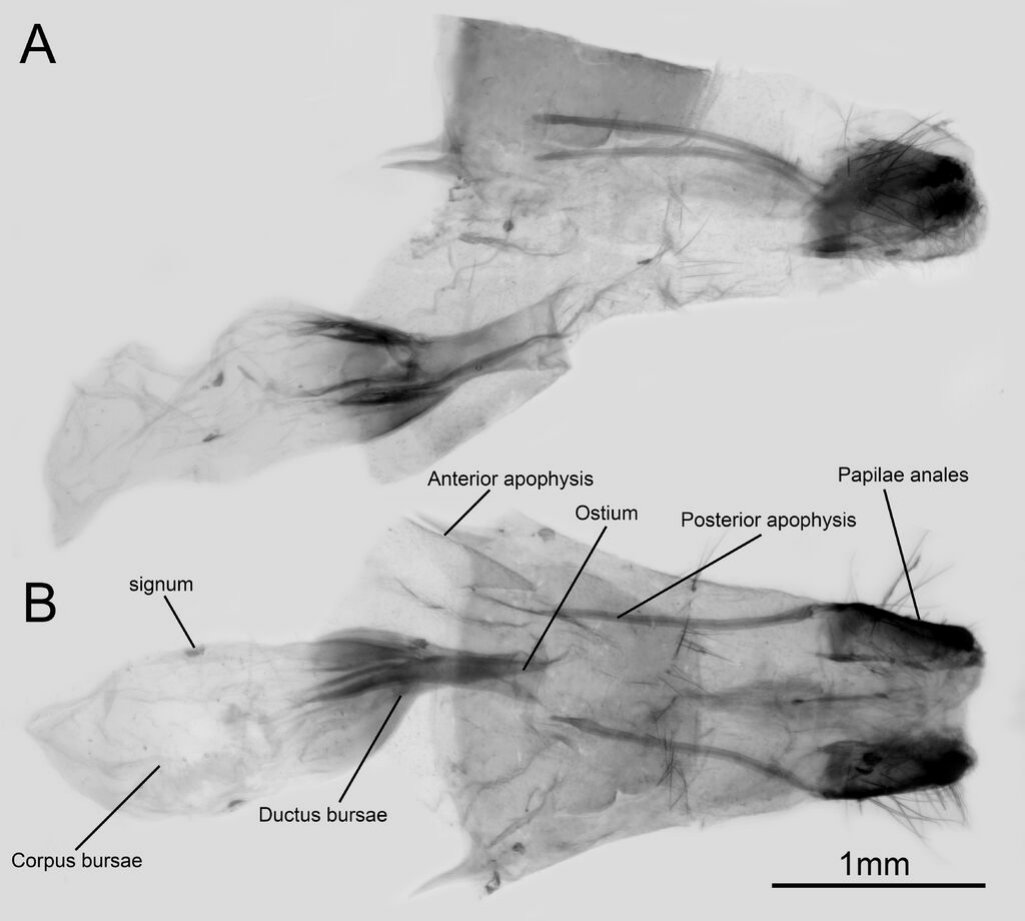
Female genitalia of *Q.qinlingana* from Sichuan: lateral view (**A**) and dorsal view (**B**).

Female genitalia (Fig. 2) with papillae anales highly sclerotised; posterior apophyses long and slender; anterior apophyses acute; ostium nearly round. Ductus bursae moderately sclerotised; corpus bursae dentoliva-shaped.

**Figure 3.**
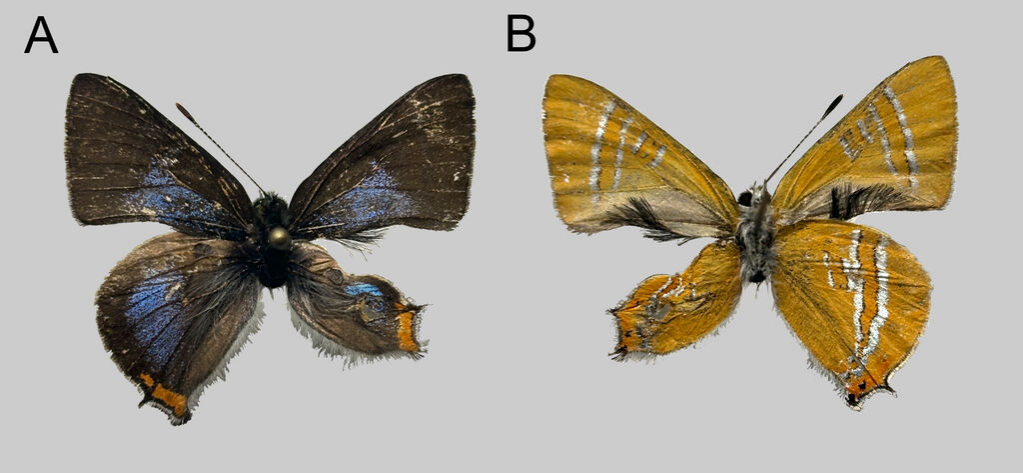
Male of *Q.qinlingana* Chou & Wang, 1995 collected in Huangguan Township, Ningshan County, Shaanxi, China: upperside (**A**) and underside (**B**).

**Male from Shaanxi Province.** Upperside (Fig. 3): Both wings dark brownish with greyish-blue iridescence. Forewing with metallic patch basally along dorsum, situated in the basal 3/5 of the space Cu_2_, 2A and basal 1/3 of Cu_1_. Hind-wing with costal margin and outer margin dark brownish. Tails short; orange tornal spot bright orange coloured. Underside (Fig. 3): Both wings pale orange coloured, post-discal band and median band pale orange with rather broad silvery-white margins. Forewing with black androconia distinctly developed on the posterior margin. Tornus of hind-wing with slight orange hue, with distinct black spots in spaces 1b and 2.

**Figure 4.**
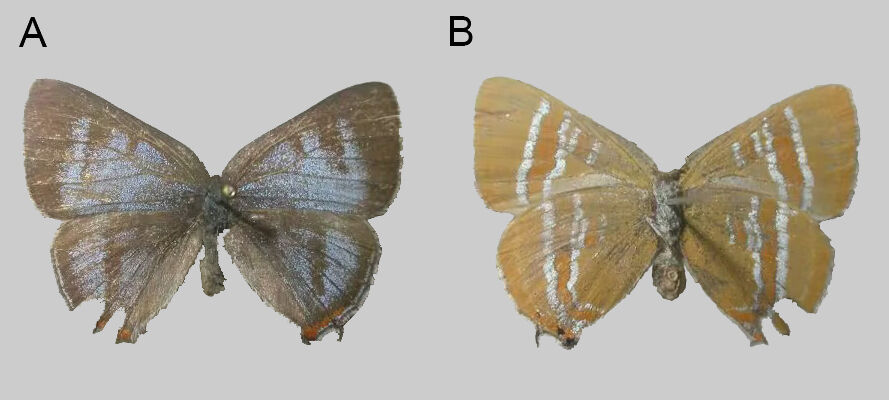
Female of *Q.qinlingana* Chou & Wang, 1995 collected in Jialing River Source Scenic Spot, Baoji City, Shaanxi, China: upperside (**A**) and underside (**B**).

**Female from Shaanxi Province** (Fig. 4). The head and antennae of the specimen are missing. Upperside (Fig. 4): Both wings with greyish-blue iridescence, forewing with veins brownish, partly suffused with blue scales; post-discal band brownish, distinctly developed; median band distinct and broad, extended to space 2. Dark brownish border broadening at apex rather developed, from costal to tornal angle. Hind-wing with costal margin and outer margin brownish; outer margin with greyish-blue marginal band. Tails short; orange tornal spot dark-coloured, present in spaces 1b, 2 and 3. Underside (Fig. 4): Both wings pale yellowish coloured, post-discal band and median band pale orange with rather broad silvery-white margins. Tails black; tornus of hind-wing with slight orange hue, with distinct black spots in spaces 1b and 2.

**Figure 5.**
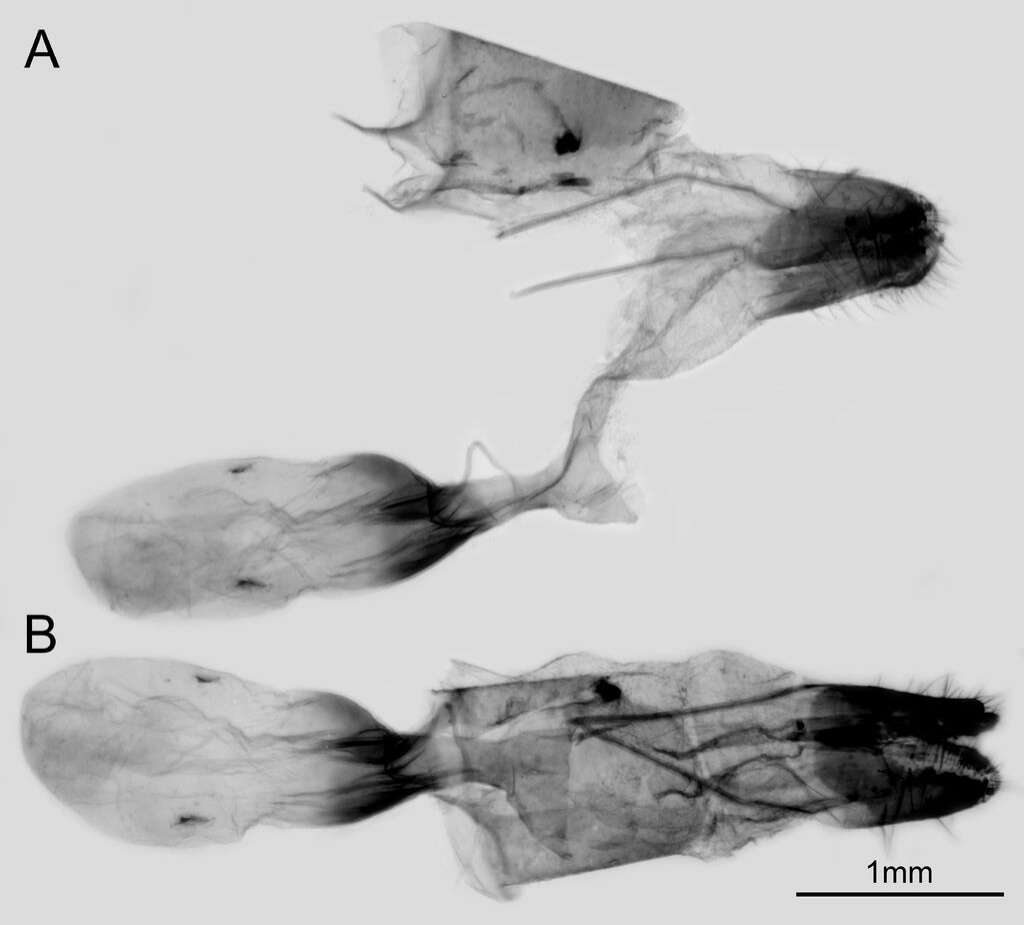
Female genitalia of *Q.qinlingana* from Shaanxi: lateral view (**A**) and dorsal view (**B**).

Female genitalia (Fig. 5) with papillae anales highly sclerotised; posterior apophyses long and slender; lamella antevaginalis and lamella postvaginalis not distinctly sclerotised; anterior apophyses acute; ostium broad and round. Ductus bursae highly sclerotised; corpus bursae dentoliva-shaped.

#### Distribution

West China (Shaanxi, Gansu, Sichuan)

#### Taxon discussion

**Figure 6.**
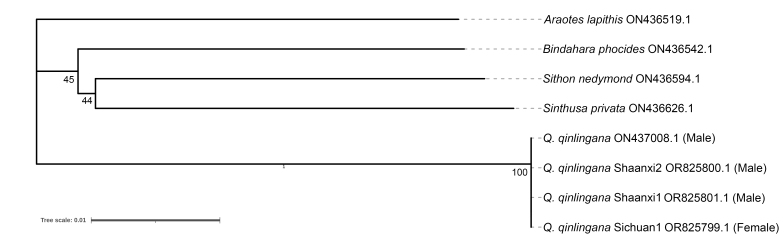
The Neighbour-Joining phylogenetic tree of the *Q.qinlingana*. Values at nodes indicate the bootstrap values.

The female specimen described in this study shows apomorphic morphological characters that prove conspecificity with the male holotype, as the forewing with veins M_1_ and R_4+5_ stalked; hind-wing with an extremely short tail and whitish or silvery-white margins of post-discal band and median band. The Neighbour-Joining phylogenetic analysis, based on partial sequences of COI (Fig. 6), also confirms that the female specimen and the male specimens belong to the same species, because the four specimens of *Q.qinlingana* were clearly defined as monophyletic with high bootstrap support and the branch length of all *Q.qinlingana* samples is 0, indicating that there is no genetic difference between samples.

#### Notes

**Figure 7.**
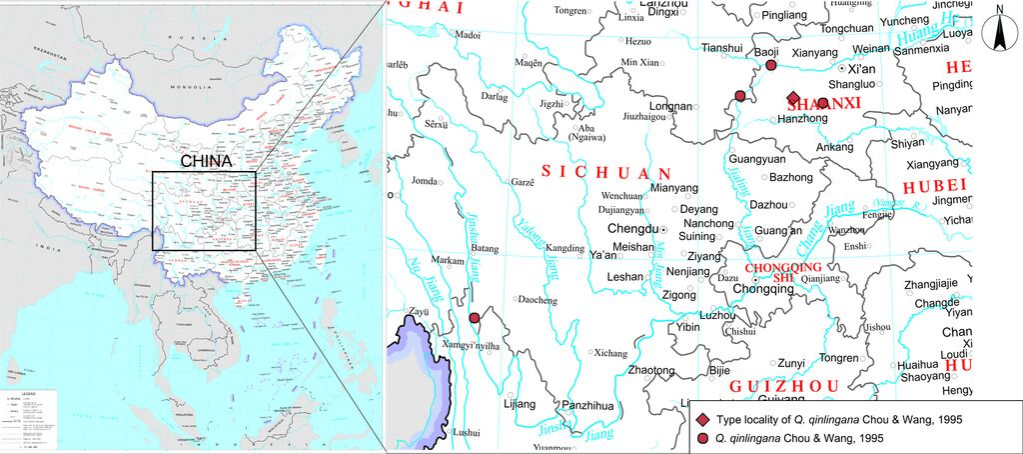
Distribution map of *Q.qinlingana* Chou & Wang, 1995. Map of China from http://bzdt.ch.mnr.gov.cn/index.html.

Before this study, there were fewer than a dozen known specimens of *Q.qinlingana* and females of the species were not described. Here, we first described the female of *Q.qinlingana* and provided its distribution information (Fig. 7). There are minor morphological differences in specimens from Sichuan and Shaanxi. The colour of wings and the shape of discal and median bands: in the specimen from Sichuan with both bands narrower, margins on the underside narrower and whitish, while in the specimen from Shaanxi, broad and silvery white. The tails: in the Sichuan specimen extremely short, but in the Shaanxi specimen, comparatively long. The tornal spot: the Sichuan specimen with tornal spot large and bright-coloured, while in the Shaanxi specimen, comparatively small and dark. However, there is no visible difference in female genitalia between the two specimens and no difference on partial sequences of COI between specimens from Shaanxi and Sichuan, so we treat them as the same taxon.

## Supplementary Material

XML Treatment for Qinorapala

XML Treatment for Qinorapala qinlingana
